# Myocardial Work Brings New Insights into Left Ventricular Remodelling in Cardio-Oncology Patients

**DOI:** 10.3390/ijerph19052826

**Published:** 2022-02-28

**Authors:** Vera Vaz Ferreira, Tania Branco Mano, Isabel Cardoso, Madalena Coutinho Cruz, Luísa Moura Branco, Luís Almeida-Morais, Ana Timóteo, Ana Galrinho, Alexandra Castelo, Pedro Garcia Brás, Diana Simão, Mariana Sardinha, António Gonçalves, Rui Cruz Ferreira

**Affiliations:** 1 Department of Cardiology, Hospital de Santa Marta, Centro Hospitalar Universitário de Lisboa Central, 1169-024 Lisbon, Portugal; taniabmano@gmail.com (T.B.M.); isagmcardoso@gmail.com (I.C.); madalena.cruz89@gmail.com (M.C.C.); luisamourabranco@me.com (L.M.B.); lmmorais88@gmail.com (L.A.-M.); ana_timoteo@yahoo.com (A.T.); anaisabelgalrinho@gmail.com (A.G.); alexandrafbcastelo@gmail.com (A.C.); pedrobras3@gmail.com (P.G.B.); antonio.a.goncalves.14@gmail.com (A.G.); cruzferreira@netcabo.pt (R.C.F.); 2 Department of Oncology, Hospital Santo António dos Capuchos, Centro Hospitalar Universitário de Lisboa Central, 1169-050 Lisbon, Portugal; verasvferreira@campus.ul.pt (D.S.); 74254@chlc.min-saude.pt (M.S.)

**Keywords:** breast cancer, cancer therapeutics-related cardiac dysfunction, myocardial work, speckle tracking imaging

## Abstract

Serial transthoracic echocardiographic (TTE) assessment of 2D left ventricular ejection fraction (LVEF) and global longitudinal strain (GLS) are the gold standard screening methods for cancer therapeutics-related cardiac dysfunction (CTRCD). Non-invasive left ventricular (LV) pressure-strain loop (PSL) provides a novel method of quantifying myocardial work (MW) with potential advantages to evaluate the impact of cardiotoxic treatments on heart function. We prospectively assessed breast cancer female patients undergoing cancer therapy through serial monitoring by 2D and 3D TTE. Patients were evaluated at T0, T1 and T2 (before, 4–6 and 12–14 months after starting therapy, respectively). Through PSL analysis, MW indices were calculated. A total of 122 patients, with a mean age of 54.7 years, who received treatment with anthracyclines (77.0%) and anti-HER2 (75.4%) were included. During a mean follow-up of 14.9 ± 9.3 months, LVEF and GLS were significantly diminished, and 29.5% developed CTRCD. All MW indices were significantly reduced at T1 compared with baseline and tended to return to baseline values at T2. Global work index and global work efficiency showed a more pronounced variation in patients with CTRCD. The presence of more than one cardiovascular risk factor, obesity and baseline left atrium volume were predictors of changes in MW parameters. In conclusion, breast cancer treatment was associated with LV systolic dysfunction as assessed by MW, with its peak at 4–6 months and a partial recovery afterwards. Assessment of myocardial deformation parameters allows a more detailed characterization of cardiac remodelling and could enhance patient screening and selection for cardioprotective therapeutics.

## 1. Introduction

Breast cancer is one of the most common cancers worldwide, representing 11.7% of all new cancer cases and 6.9% of all deaths from cancer in 2020 [1]. Substantial advances in cancer therapies in the last decades have reshaped the prognosis of cancer patients [2]. However, increased survival along with aging of the cancer population, which is associated with a greater burden of co-morbidities, has been accompanied by a rise in adverse cardiovascular complications, particularly when there are pre-existing cardiovascular diseases [3,4,5]. Because of that, the incidence of cardiotoxicity continues to grow, which can compromise the effectiveness of cancer therapy [2].

Cancer therapeutics-related cardiac dysfunction (CTRCD) is relatively common in breast cancer treatment, both early and late after treatment. Classically, CTRCD was described as a consequence of anthracycline therapy, although other oncology therapies, such as human epidermal growth factor receptor 2 (HER2) inhibitors, tyrosine kinase inhibitors and proteasome inhibitors, are currently recognized as potential causes as well [2]. The overall incidence of cardiotoxicity has been reported in up to 26% of patients receiving anthracyclines standard cumulative doses [4]. Thus, cardiac surveillance is highly recommended in these patients to enable prevention and treatment of cardiotoxicity [5,6]. Standard pharmacological therapies indicated for the treatment of heart failure have been shown to be also useful in CTRCD [2].

Two-dimensional (2D) left ventricle ejection fraction (LVEF) has been the most widely used tool to diagnose cardiac dysfunction in this setting, although several shortcomings, such as the need for geometrical assumptions, apical foreshortening, load dependency and measurement variability have been described [7,8]. Three-dimensional (3D) LVEF has a greater reproducibility of sequential assessments and a better correlation with cardiac magnetic resonance imaging [9]. More recently, 2D speckle-tracking echocardiography (STE) has been associated with an improvement in the understanding of the cardiac performance, providing earlier and more sensitive detection of left ventricle (LV) dysfunction when compared with LVEF [10,11,12], even in the context of normal LVEF [13,14]. Impaired global longitudinal strain (GLS) is independently associated with increased incidence of CTRCD [2,15] and has been validated as a quantitative assessment tool in the surveillance of these patients during and after cancer therapy [2], although load dependency is one of the key limitations of LV longitudinal strain (LS), which can constrain the evaluation of LV performance under different hemodynamic conditions and when undergoing serial monitoring. Myocardial work (MW) is a novel non-invasive technique to assess myocardial performance. This tool supplies a LV pressure-strain loop (PSL) by examining both LV deformation and afterload, which incorporates non-invasively measured arterial blood pressure and LS acquired by STE analysis, providing an accurate assessment even in cases of changes in the afterload [16,17].

To the best of our knowledge, there are no published data regarding the effects of breast cancer therapy on MW parameters. The assessment of MW may provide further insights into the process of LV remodelling after cancer treatment.

The aims of this study were to prospectively assess LV MW before and after cancer treatment, including anthracycline and/or anti-HER therapy, in a real-world cohort of female breast cancer patients and to determine predictors of MW variation.

## 2. Materials and Methods

This study is a part of a single-centre project (INV 308) that was approved by the ethics committee of the involved institution (Centro Hospitalar Universitário de Lisboa Central—Nº1199). The investigation conformed to the principles outlined in the Helsinki Declaration. Informed consent was provided from all subjects involved in the study.

### 2.1. Study Participants

Analysis of consecutive patients with breast cancer undergoing cancer treatment that were prospectively included in an echocardiographic surveillance protocol from January 2018 to December 2020.

Patients undergoing serial transthoracic echocardiograms, including standard parameters [8], 2D GLS and blood pressure measurement, with baseline, 4–6 months and 12–14 months evaluation, were selected for this analysis.

Patients with prior cancer treatment (chemotherapy, anti-HER2 or chest-directed radiotherapy), previous myocardial infarction, coronary re-vascularization, significant valvular heart disease, atrial fibrillation/flutter or pacemaker and inadequate endomyocardial border definition for LV LS measurements (more than two segments) were excluded.

Demographic data, anti-cancer therapy and echocardiographic parameters were prospectively recorded. Cumulative doses of each anthracycline were converted to doxorubicin equivalents by considering drug potency, using acknowledged methods [18].

Hypertension (according to European Society of Cardiology guidelines (ESC) guidelines [19], with or without anti-hypertensive drugs), diabetes (according to ESC guidelines [20], with or without anti-diabetic drugs), obesity (body mass index above 30 Kg/m^2^) and smoking history (past smoking history or active smoking) were the studied cardiovascular risk factors.

### 2.2. Echocardiographic Acquisition and Processing

All included patients underwent an echocardiographic evaluation before (T0), at 4–6 months (T1) and at 12–14 months (T2) from the initiation of cancer therapy. Additional echocardiographic assessments were performed at the discretion of the oncologist. All echocardiograms were performed by a single, experienced cardiologist unblinded to the previous exams. A complete standard echocardiographic study was performed using commercially available system (Vivid E95™; GE Healthcare). Echocardiographic assessment was performed according to the American Society of Echocardiography/European Association of Cardiovascular Imaging’s recommendations [8,21].

The 2D LVEF was calculated according to the biplane Simpson’s method. The 3D LVEF and volumes evaluation were performed with real-time, full-volume data acquisition by a 3D volumetric transducer in apical view of the LV. Biplane algorithm was used to calculate LA volume, which includes the 4-chamber and 2-chamber apical views [8].

**Figure 1:**
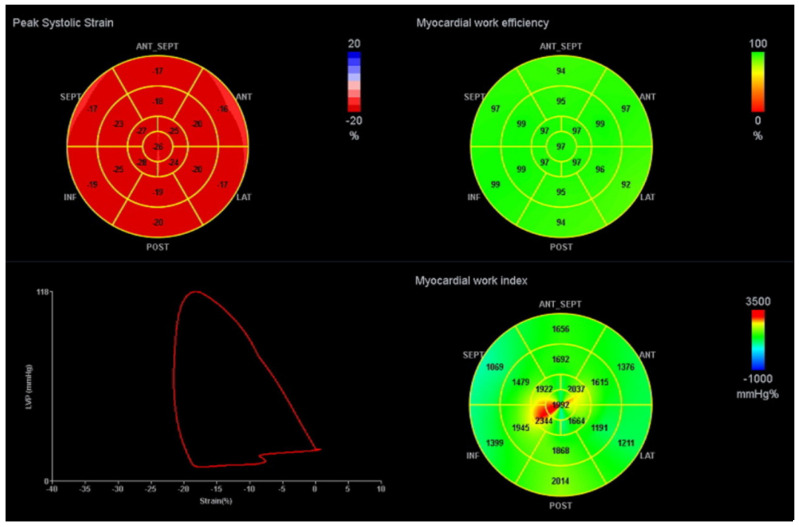
Example of a LV PSL and MW indices. The red curve represents the LV PSL. The bull’s-eye plot on the left represents peak systolic strain; the upper right shows GWE and bottom right GWI. ANT: anterior, ANT_SEPT: antero-septal, INF: inferior, LAT: lateral, POST: posterior, SEPT: septal. GWI: global work index, GWE: global work efficiency.

A commercially available software for offline data analysis in a workstation (EchoPAC BT12 workstation, GE Healthcare) was used. 2D LS was assessed using the STE technique with semi-automatic tracing of the LV endocardium, after manual demarcation of the mitral valve edges and apex. 2D GLS was determined as the average of the segmental peak strain in 17 segments [8]. GLS was not estimated if more than two segments were rejected. The indexes of MW were calculated, taking into account 2D STE and estimated LV pressure. The peak systolic LV pressure was estimated based on the systolic blood pressure measured non-invasively in the brachial artery at rest in the supine position, assuming the absence of LV outflow obstruction. A LV PSL was then constructed automatically. The ejection and isovolumetric phases of the LV PSL were defined based on the opening and closure of the mitral and aortic valves. The total work within the area of the LV PSL during the LV ejection period was defined as the global myocardial work index (GWI). The MW corresponding to the period of segmental shortening was defined as global constructive work (GCW), and the MW corresponding to the period of segmental elongation was defined as wasted work (GWW). Regarding isovolumetric relaxation, MW during shortening was defined as GWW, and MW during lengthening was defined as GCW. GCW and GWW were calculated as the averages of the 17 segmental values. Global work efficiency (GWE) was obtained as GCW/(GCW + GWW) × 100%—Figure 1. A cohort of healthy subjects with vendor-specific values were used as reference values for comparison with our sample [22].

### 2.3. CTRCD Definition

CTRCD was defined as an absolute decrease in 2D or 3D LVEF of >10% to a value <54% or a relative decrease in 2D GLS of >15%, according to ESC [2] and the EACVI consensus document on cardiovascular imaging in cancer patients [23]. By considering both LVEF and GLS data, the definition of CTRCD is not only sensitive but also associated with prognosis in such a clinical scenario [11,12,24].

### 2.4. Statistical Analysis

Continuous variables were expressed as mean ±SD when normality was verified or as median (interquartile range (IQR)) when normality was not verified (Shapiro–Wilk or Kolmogorov–Smirnov tests). Categorical data are presented as frequency (percentage). Continuous variables were analysed using student’s T-test or the Mann–Whitney test when the normality was not verified. Pairing of baseline characteristics and outcomes was performed using a chi-square test or Fisher’s exact test for categorical variables.

Follow-up echocardiograms were compared with baseline for detection of changes in 2D and 3D echocardiographic parameters. CTRCD was considered if the above-mentioned criteria were encountered in T1 or T2, compared to the baseline assessment. Pearson’s correlation was applied to assess correlations between continuous variables. Univariable logistic regression analysis was used to identify which variables predicted the variation of MW parameters. Significance was set at α = 0.05. Statistical analysis was performed using dedicated software (SPSS, version 25.0, IBM SPSS).

## 3. Results

### 3.1. Baseline Characteristics of Participants and Cancer Therapy

Of a total sample of 150 patients, 28 were excluded due to previous cancer treatment (9), significant valvular disease (4), atrial fibrillation (3), and inadequate endomyocardial border definition for LS measurements by 2D STE (12). A total of 122 women were assessed during a mean follow-up of 14.9 ± 9.3 months (Table 1). Mean age at first echocardiogram was 54.7 ± 12.0 years. Most patients (67.2%) had at least one cardiovascular risk factor. The majority was treated with anthracyclines (77.0%), with a mean cumulative dose of 268.6 ± 71.8mg/m^2^; 75.4% of patients were submitted to HER2 inhibitor therapy and 77.0% to radiotherapy. In 51.6% of cases, anthracyclines regimens were followed by one year of trastuzumab every 21 days in the dose of 6 mg/Kg, and 24.6% underwent dual anti-HER2 blockade with trastuzumab and pertuzumab. Mean chemotherapy and anti-HER2 duration were 4.9 (IQR 3.5–5.4) months and 12.6 (IQR 11.9–16.7) months, respectively. There was no overlap between anthracyclines and anti-HER2 treatment.

### 3.2. CTRCD on Anthracyclines and Anti-HER2 Therapy

Table 2 shows the results of TTE analysis at the three evaluation moments. LV end-diastolic, end-systolic and left atrium (LA) volumes were significantly increased at T1. The 2D and 3D LVEF were significantly reduced during cancer treatment (2D LVEF 64.2 ± 7.6% vs. 61.1 ± 8.2%, *p* = 0.006, and 3D LVEF 60.2 ± 6.7% vs. 56.9 ± 6.3%, *p* = 0.022, comparing T0 and T1). The 2D GLS was also more impaired at T1 (−19.8 ± 2.7% vs. −18.5 ± 3.0%, *p* = 0.003). During follow-up, 36 (29.5%) patients developed CTRCD. Of these, 50% were diagnosed according to LVEF criterion. Eight (6.6%) patients were temporarily dropped out of cancer treatment because of cardiotoxicity, mean LVEF 48.3% ± 6.8%, while on anti-HER2 therapy, and half of these patients had previous therapy with anthracyclines. Ten patients (9.1%) started cardioprotective therapy (angiotensin-converting enzyme inhibitors or angiotensin receptor blocker in 4, betablockers in two and both in four patients) during chemotherapy due to asymptomatic cardiotoxicity, that is, due to a decrease in 2D or 3D LVEF >10% to a value <54% or a relative decrease in 2D GLS >15%.

### 3.3. MW Indices

MW indices of the study population are shown in Table 2. At T1, patients showed significantly lower values of GWI (1756.9 ± 319.2 mmHg% vs. 1614.3 ± 338.5 mmHg%, *p* = 0.005) and GCW (GCW 2105.6 ± 352.0 vs. 1970.5 ± 376.2 mmHg%, *p* = 0.015) as well as higher values of GWW (121.1 ± 66.6 vs. 161.1 ± 84.1 mmHg%, *p* = 0.001) compared with baseline assessment. This resulted in significantly lower GWE at T1, with a mean of 91.1 ± 4.5% compared with 93.5 ± 3.1% at T0 (*p* = 0.001). According to recent published reference values [20], 11.4% and 17.1% had baseline GWI and GWE values below the limit of normality for female, which increased to 20.2% and 36.9% at T1, respectively (Table 3). Similarly to LVEF and GLS, the MW parameters improved from T1 to T2, approaching baseline values, although not reaching statistical significance.

**Figure 2:**
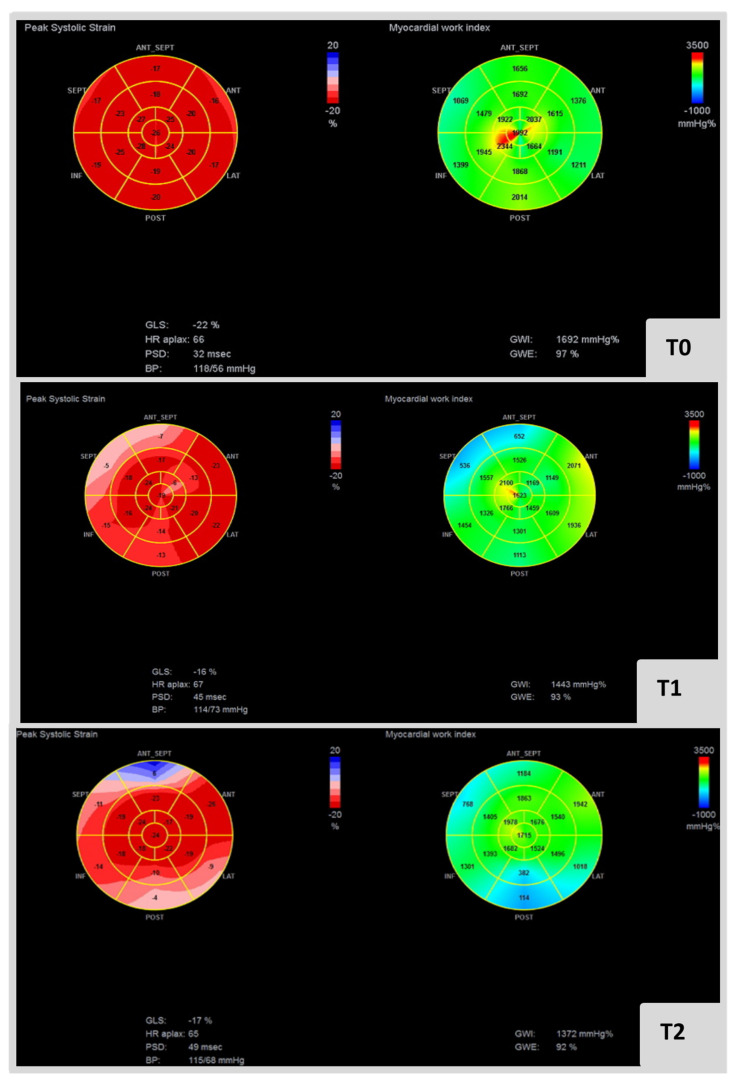
Bull eye’s plot at left and right represent GLS and GWI at T0, T1 and T2, respectively, in cardio-oncology patient submitted to anthracyclines and anti-HER2 therapy for breast cancer. It is also specificized blood pressure and GWE in each analysis. GWI: global work index, GCW: global constructive work, GWW: global wasted work, GWE: global work efficiency.

Figure 2 presents an example of MW indices in a cardio-oncology patient submitted to anthracyclines and anti-HER2 therapy for breast cancer.

### 3.4. Comparison of Patients with and without CTRCD

Anti-HER2 duration treatment, including anti-HER2 regimen longer than 12 months, was associated with CTRCD (*p* = 0.042 and *p* = 0.018, respectively). Other clinical features, chemotherapy regimens and echocardiographic data did not significantly differ in patients with or without CTRCD.

Patients presenting CTRCD revealed a significant decrease in GWI and GWE at T1 compared with women without CTRCD (GWI 1.8 ± 21.6 vs. −14.2 ± 18.5%, *p* = 0.004 and GWE −1.0 ± 3.0 vs. −3.6 ± 3.9%, *p* = 0.005). GWW tended for a substantial increase at T1 in patients with cardiotoxicity (27.6 ± 76.3% vs. 64.1 ± 68.0%, *p* = 0.051—Table 4).

Patients with MW indexes below the reference values at T0 did not present an increased risk of CTRCD during follow-up (Table S1—Supplementary Materials).

### 3.5. Predictors of MW Worsening

The univariate analysis assessing the parameters associated with MW indices variation is presented in Table S2 (Supplementary Materials). Obesity was a predictor of MW worsening when comparing T1 with T0, including GWI (β −18.226, 95%CI −30.512 to −5.939, *p* = 0.004), GWE (β −2.976, 95%CI 4.949 to −1.004, *p* = 0.004) and GCW (β −15.190, 95%CI −26.426 to −3.955, *p* = 0.009). Hypertension at baseline was associated with significant GWI worsening at T1 (β −12.160, 95%CI −22.992 to −1.328, *p* = 0.028). The presence of more than one cardiovascular risk factor was correlated with worsening of GWE at T1 (β −1.849, 95%CI −3.645 to −0.054, *p* = 0.044). Increased LA volumes at baseline were associated with worsening of GWI and GCW values from baseline to T1 (β −0.380, 95%CI −0.726 to −0.034, *p* = 0.032 and β −0.356, 95%CI −0.676 to 0.037, *p* = 0.029, respectively).

## 4. Discussion

In the present study, we prospectively evaluated MW indices prior to, 4–6 months and 12–14 months after cancer therapy in 122 consecutive breast cancer patients.

The main findings of the present study can be summarized as follows:

1. Breast cancer patients under cancer therapy showed impaired values of global LV myocardial work parameters—GWI, GCW, GWW and GWE—compared to baseline;
2. Twelve months after starting therapy, MW indices tended to return to baseline values, although not completely;
3. More than one cardiovascular risk factor, obesity and baseline LA volume were predictors of MW parameters variation.

Although breast cancer survival continues to improve, persistent injury to myocardium resulting from anthracycline and anti-HER2 toxicity may lead to higher non-cancer-related morbidity and mortality [25,26]. Anthracycline induced cardiotoxicity is caused by multiple mechanisms resulting in myocardial cell death and interstitial fibrosis immediately after exposure and is dependent on the cumulative dose [27]. It is also associated with irreversible cardiac damage and progressive cardiac remodelling as a late consequence [2,15]. In contrast to anthracyclines, cardiac dysfunction expressed during anti-HER2 treatment occurs due to cell dysfunction rather than loss of myocytes and is potentially reversible [15].

In CTRCD, the reversibility of cardiac dysfunction is inversely related to the time from the start of chemotherapy [28,29]. The ideal screening method of CTRCD should be able to detect subclinical LV dysfunction and therefore prompt an early initiation of neurohumoral therapy, preventing the progression to overt heart failure and chemotherapy suspension. Current treatment consensus suggests an interval of three to six months before monitoring cardiac function [2,30,31].

Most of our study population received anthracycline chemotherapy with a mean dose of 268.6 ± 71.8 mg/m^2^. We report an incidence of CTRCD that is higher than most previous studies with anthracyclines for the described cumulative dose [2]. Within the current cohort, changes indicative of LV sub-clinical dysfunction using GLS were observed in 50% of CTRCD patients, without any evidence of cardiotoxicity by LVEF. As the definition of CTRCD changes towards a more sensitive one, it is expected that its incidence will be higher than in prior studies applying LVEF instead of 2D GLS and LVEF [32]. Another factor that may have probably contributed to the relatively high incidence of CTRCD in our sample was the frequent use of anti-HER2 agents, cyclophosphamide and chest radiotherapy, which are known to have an additive effect to anthracyclines [33]. Duration of HER2 inhibitor therapy was a determinant of CTRCD in our population, a finding previously reported in the literature [34].

The incidence of myocardial damage after anthracycline chemotherapy may be enhanced by smoking [35], pre-existing cardiovascular disease or individual patient genetic predisposition [25]. This background possibly had impact on CTRCD incidence, even though the development of CTRCD in the current study was not associated with any individual cardiovascular risk factors or clinical parameters. In addition, despite the age and high prevalence of cardiovascular risk factors, patients with a prior diagnosis of CAD (prior MI or re-vascularization) were excluded, and included patients did not present clinical, electrocardiographic or echocardiographic signs of ischemic heart disease.

In 2D echocardiography, conventional LVEF lacks sensitivity for detecting subtle myocardial dysfunction, and thus, STE has been introduced [13,14]. A number of studies have demonstrated the diagnostic and predictive utility of LS measures in detecting cardiotoxicity [2,29,30]. A systematic review described that changes in LS occurred prior to significant variations in LVEF, and GLS was the most consistent measure, whilst LVEF had variability with clinically significant decline noticeable only at late follow-up [36]. More recently, a GLS-based approach to cardiac surveillance, followed by initiation of cardioprotective therapy, showed to decrease the degree of reduction in LVEF and the incidence of CTRCD at 1-year follow-up [24].

The present study revealed significantly reduced values of LVEF and GLS during follow-up of anthracycline-based chemotherapy and during the course of trastuzumab treatment. However, LVEF changes remained in the normality range and tended to recover at 12 months after starting cancer treatment. Possibly, if follow-up was extended, anti-HER2 therapy would be interrupted in a substantial percentage, and LV function could be completely reversible, according to previous studies [33,34,35]. However, patients who need to continue therapy still present an increased risk of CTRCD.

A reduction of LV LS ≥ 15% is the cut-off defined for myocardial dysfunction post anti-cancer therapies in the ESC position paper [2], although this cut-off may not provide satisfactory sensitivity for the early detection of CTRCD. In addition, LVEF [37] and myocardial deformation indices are relatively load-dependent [16,38], which might represent a limitation in case of frequent changes in the hemodynamic conditions of cancer patients [39]. Hence, additional echocardiographic parameters are being studied as potential tools to assess the LV performance in patients undergoing cancer therapy. MW reflects LV function, integrating simultaneously LV deformation and LV afterload data.

Russell et al. [17] corroborated this technique against invasive LV pressure measurements, and LV PSL area exhibited a strong correlation with myocardial metabolism when evaluated on positron emission tomography. Several studies have already applied MW indices to different cardiac conditions [40,41,42,43,44,45,46].

To the best of our knowledge, this is the first study concerning the effects of cancer treatment on MW indices. The use of LV PSL in this population demonstrates that LV mechanics are affected in all MW components during oncology treatment. This is concordant with the evidence obtained with 2D LVEF and 2D GLS [47], as well as with more recent reports of 3D STE [48,49,50].

Interestingly, baseline GWI and GWE were below the reference values in a proportion of women [22], possibly showing a selection of high-risk patients with cardiovascular risk factors. Abnormal values of 2D strain parameters in cancer patients before treatment initiation have previously been reported [51,52], suggesting a direct effect of cancer on cardiac mechanics, which eventually can also justify these values on MW indices. However, in our sample, MW indices below the reference values at baseline were not associated with an increased risk of CTRCD.

Obesity, the presence of more than one cardiovascular risk factor and increased baseline LA volume were univariate predictors of significant MW indices worsening. Pre-existing cardiovascular risk factors and anatomic alteration of LA probably reflected previous sub-clinical LV dysfunction or more susceptibility to develop it when submitted to toxic agents.

In our population, GWI and GWE had a more pronounced variation in patients with CTRCD. This variation can have clinical impact not yet studied, possibly identifying a high-risk sub-group of patients that might benefit of closer monitoring and earlier initiation of cardioprotective therapies, even before LVEF impairment. Future recommendations could also rely on screening of a combination of MW indices and cardiac biomarkers.

Further studies are needed to determine whether a MW-guided approach reduces the long-term risk of heart failure and improves clinical outcomes.

## 5. Study Limitations

This study has some limitations that should be acknowledged. A potential selection bias cannot be excluded, as these patients were referred by assistant oncologists. The relatively short follow-up of this sample may have contributed to an underestimation of CTRCD. Moreover, this was a single-centre study, and the results may not be applicable to other settings.

Since a considerable percentage of patients received a combined treatment, including anthracyclines, HER2 inhibitor therapy and/or radiotherapy, it is difficult to assess the individual contribution of each therapy for inducing CTRCD, which may vary between different regimens. Consequently, the results cannot be generalized to mono-therapy anthracycline regimens.

The prognostic value of MW has been reported in different clinic sub-sets, such as hypertrophic cardiomyopathy and in LV desynchrony before cardiac re-synchronization therapy. It is likely that worsening of MW parameters also conveys prognostic information in patients with cancer undergoing chemotherapy, although such hypothesis needs to be demonstrated. Similarly, whether an earlier initiation of cardioprotective therapies, as guided by MW data, is associated better outcomes needs to be studied.

Another limitation concerns the existing overlap that may exist between echocardiographic parameters that evaluate LV contractile or systolic function in every study addressing the natural history of CTRCD.

In addition, MW indices reference values still not entirely defined, and inter-vendor discrepancies have also been noticed. In our study, we compared our values with vendor-specific values of a cohort of healthy subjects [20]. Therefore, direct comparison of results obtained by different scanners should be made with caution.

## 6. Conclusions

Left ventricular contractility, as assessed by MW, worsened during anthracycline and anti-HER therapy for breast cancer and tended to return to baseline after cessation of therapy. The integration of MW assessment in the routine echocardiographic surveillance of patients with breast cancer undergoing cancer therapy might be considered in order to offer a targeted monitoring with an early detection of LV dysfunction and allow for a timely initiation of cardioprotective treatment.

## Figures and Tables

**Table 1:** Baseline characteristics and cancer treatment during the study.

Baseline Characteristics	N = 122
Clinical history	
Age (years)	54.7 ± 12.0
Hypertension	41 (33.6%)
Diabetes mellitus	9 (7.4%)
Hypercholesterolemia	37 (30.3%)
Smoking history	30 (24.6%)
Obesity	20 (17.4%)
Body mass index (kg/m^2^)	26.7 ± 4.2
Cardiovascular risk factors	
None	40 (32.7%)
1	42 (34.4%)
≥2	40 (32.7%)
Previous medication with ACE inhibitors/ARBs	30 (24.8%)
Previous medication with beta-blocker	15 (12.4%)
Subtypes of breast cancer	
ER + HER2-	20 (16.4%)
ER + HER2+	56 (45.9%)
ER-HER2-	10 (8.2%)
ER-HER2+	36 (29.5%)
Therapy regimen	
Neoadjuvant chemotherapy	47 (38.5%)
Adjuvant chemotherapy	81 (66.4%)
Anthracyclines	94 (77.0%)
Doxorubicin	60 (49.2%)
Epirubicin	34 (27.9%)
Cumulative dose (mg/m^2^)	268.6 ± 71.8
Anti-HER2	92 (75.4%)
Trastuzumab	91 (74.6%)
Pertuzumab	32 (26.2%)
T-DM1	14 (11.5%)
Cyclophosphamide	93 (76.9%)
Taxane	114 (93.4%)
Anthracyclines and anti-HER2	63 (51.6%)
Median chemotherapy time (months)	4.9 (3.5–5.4)
Median Anti-HER2 time (months)	12.6 (11.9–16.7)
Chest radiation therapy	94 (77.0%)
Median total dose (Gy)	50 (50–60)

ACE: angiotensin-converting enzyme, ARB: angiotensin II receptor blocker, ER: oestrogen receptor; HER2: human epidermal growth factor receptor, TDM-1: trastuzumab emtansine. Data are expressed as mean ± SD, median (IQR) or number (percentage).

**Table 2:** Echocardiographic parameters before and during chemotherapy.

TTE Variable	T0	T1	T2	*p*-Value (T0 vs. T1)	*p*-Value (T1 vs. T2)	*p*-Value (T0 vs. T2)
2D parameters						
LV end-diastolic volume (mL)	75.1 ± 19.0	82.9 ± 20.2	78.9 ± 18.6	0.005	0.122	0.137
LV end-systolic volume (mL)	27.0 ± 10.0	32.5 ± 12.2	30.5 ± 11.2	0.001	0.204	0.019
LVEF (%)	64.2 ± 7.6	61.1 ± 8.2	61.6 ± 8.0	0.006	0.656	0.016
GLS (%)	−19.8 ± 2.7	−18.5 ± 3.0	−18.7 ± 3.1	0.003	0.686	0.012
LV stroke volume (mL)	68.8 ± 15.0	70.6 ± 18.6	66.1 ± 14.6	0.538	0.019	0.292
LV cardiac output (L/min)	5.3 ± 1.5	5.3 ± 1.4	4.9 ± 1.2	0.943	0.082	0.091
LA diameter (mm)	36.2 ± 4.6	33.3 ± 4.9	37.0 ± 5.4	0.892	0.355	0.280
LA volume (mL)	44.4 ± 14.8	50.3 ± 14.1	48.6 ± 15.1	0.007	0.424	0.049
Transmitral E/A ratio	1.1 ± 0.4	1.0 ± 0.4	1.0 ± 0.3	0.711	0.289	0.139
Mitral E/e’ ratio	8.1 ± 2.5	8.3 ± 2.6	8.6 ± 2.9	0.683	0.491	0.277
TAPSE (mm)	22.5 ± 3.7	22.5 ± 3.5	22.9 ± 4.0	0.990	0.532	0.546
Tricuspid S’ (cm/s)	12.7 ± 2.6	12.4 ± 2.5	12.2 ± 2.6	0.394	0.729	0.232
3D parameters						
LV end-diastolic volume (mL)	81.8 ± 18.5	91.4 ± 18.8	84,2 ± 18.8	0.017	0.079	0.545
LV end-systolic volume (mL)	32.8 ± 10.6	39.8 ± 11.7	34.9 ± 9.8	0.005	0.046	0.332
LVEF (%)	60.2 ± 6.7	56.9 ± 6.3	58.7 ± 5.5	0.022	0.166	0.271
Myocardial work indices						
GWI (mmHg%)	1756.9 ± 319.2	1614.3 ± 338.5	1650.6 ± 357.5	0.005	0.465	0.035
GCW (mmHg%)	2105.6 ± 352.0	1970.5 ± 376.2	2013.3 ± 379.3	0.015	0.427	0.086
GWW (mmHg%)	121.1 ± 66.6	161.1 ± 84.1	148.0 ± 85.0	0.001	0.281	0.02
GWE (%)	93.5 ± 3.1	91.1 ± 4.5	92.0 ± 4.7	0.001	0.171	0.012
Systolic blood pressure (mmHg)	120.9 ± 14.6	120.0 ± 14.4	121.8 ± 15.5	0.669	0.403	0.690

2D: two-dimensional, LV: left ventricle, LVEF: left ventricle ejection fraction, GLS: global longitudinal strain, LA: left atrium, 3D: three-dimensional, TAPSE: tricuspid annular systolic excursion, GWI: global work index, GCW: global constructive work, GWW: global wasted work, GWE: global work efficiency. Data are expressed as mean ± SD.

**Table 3:** MW indices reference values [22].

MW Indices Reference Values	Limits of Normality	Below at T0 (%)	Below at T1 (%)
GWI (mmHg%)	<1310	11.4	20.2
GCW (mmHg%)	<1543	10.5	15.5
GWW (mmHg%)	>278	5.7	10.7
GWE (%)	<90	17.1	36.9

GWI: global work index, GCW: global constructive work, GWW: global wasted work, GWE: global work efficiency.

**Table 4:** MW indices variation in the different echocardiographic assessments according to the presence or absence of CTRCD.

TTE Variable	No CTRCD	CTRCD	*p*-Value
GWI T1-T0 (%)	1.8 ± 21.6	−14.2 ± 18.5	0.004
GWI T2-T0 (%)	−1.9 ± 19.5	−14.9 ± 20.6	0.010
GCW T1-T0 (%)	−0.7 ± 19.8	−8.6 ± 17.9	0.099
GCW T2-T0 (%)	−3.0 ± 16.9	−8.4 ± 19.2	0.194
GWW T1-T0 (%)	27.6 ± 76.3	64.1 ± 68.0	0.057
GWW T2-T0 (%I	−3.1 ± 50.9	10.1 ± 62.3	0.105
GWE T1-T0 (%)	−1.0 ± 3.0	−3.6 ± 3.9	0.005
GWE T2-T0 (%)	−0.86 ± 3.1	−3.2 ± 6.7	0.053

CTRCD: Cancer therapeutics-related cardiac dysfunction, GWI: global work index, GCW: global constructive work, GWW: global wasted work, GWE: global work efficiency. Data are expressed as mean ± SD.

## Data Availability

The data presented in this study are available on request from the corresponding author. The data are not publicly available due to personal data protection.

## Supplementary Materials

The following supporting information can be downloaded at: https://www.mdpi.com/article/10.3390/ijerph19052826/s1, Table S1: Association of MW indices at baseline (T0) and CTRCD; Table S2: Association of clinical characteristics, therapy regimen and echocardiographic data with MW variation at T1 from baseline.
