# Supplementary material for: Myocardial Work Brings New Insights into Left Ventricular Remodelling in Cardio-Oncology Patients
Source: Int J Environ Res Public Health. 2022 Feb 28;19(5):2826. doi: 10.3390/ijerph19052826 (PMC8910703; doi:10.3390/ijerph19052826)
Supplement: Supplementary file 1 [file ijerph-19-02826-s001.zip › ijerph-1538761-supplementary.pdf]

Table S1 Association of MW indices at baseline (T0) and CTRCD

MW indices at T0	CTRCD	p-value
GWI <1310 mmHg%	60.0%	0.251
GWI ≥1310 mmHg%	33.7%	
GCW <1543 mmHg%	50.0%	0.439
GCW ≥1543 mmHg%	34.1%	
GWW >278mmHg%	50.0%	0.533
GWW ≤278mmHg%	34.5%	
GWE <90%	50.0%	0.366
GWE ≥90%	33.8%	

GWI: global work index, GCW: global constructive work, GWW: global wasted work, GWE: global work efficiency.

Table S2 Association of clinical characteristics, therapy regimen and echocardiographic data with MW variation at T1 from baseline

	GWI			GWE			GCW			GWW		
	β	95% CI	p-value	β	95% CI	p-value	β	95% CI	p-value	β	95% CI	p-value
Baseline characteristics												
Age (years)	0.123	-0.289 to 0.536	0.553	-0.039	-0.104 to 0.026	0.239	0.210	-0.152 to 0.572	0.252	0.291	-1.146 to 1.729	0.687
Hypertension	-12.160	-22.992 to -1.328	0.028	-0.980	-2.789 to 0.829	0.284	-8.949	-18.675 to 0.777	0.071	5.462	-33.051 to 43.976	0.778
Diabetes mellitus	-10.662	-30.782 to 9.538	0.297	-0.659	-3.961 to 2.643	0.692	-4.523	-22.533 to 13.488	0.618	-9.583	-19.374 to 60.208	0.785
Hypercholesterolemia	4.576	-6.736 to 15.887	0.423	0.010	-1.839 to 1.859	0.991	6.016	-3.973 to 16.004	0.234	-0.887	-39.940 to 38.166	0.964
Obesity	-18.226	-30.512 to -5.939	0.004	-2.976	-4.949 to -1.004	0.004	-15.190	-26.426 to -3.955	0.009	19.144	-25.681 to 63.909	0.397
Smoking history	-6.806	-19.124 to 5.511	0.274	-0.633	-2.649 to 1.382	0.533	-5.061	-16.027 to 0.905	0.361	37.781	-3.955 to 79.518	0.075
More than one cardiovascular risk factor	-10.173	-21.275 to 0.929	0.072	-1.849	-3.645 to -0.054	0.044	-6.734	-16.696 to 3.229	0.182	20.077	-18.682 to 58.837	0.305
Previous medication with ACE inhibitors/ARBs	-3.604	-15.104 to 7.896	0.534	-1.753	-3.582 to 0.076	0.060	0.550	-9.689 to 10.790	0.915	37.036	-1.600 to 75.671	0.060
Previous medication with beta-blocker	-5.062	-19.949 to 9.825	0.500	-1.500	-3.903 to 0.904	0.217	-4.481	-17.700 to 8.739	0.501	22.746	-28.296 to 73.787	0.377
Therapy regimen												
Neoadjuvant chemotherapy	-7.905	-18.282 to 2.472	0.133	-1.311	-3.001 to 0.379	0.126	-1.960	-11.329 to 7.409	0.678	48.529	14.163 to 82.894	0.006
Adjuvant chemotherapy	2.184	-8.854 to 13.221	0.694	0.620	-1.066 to 2.307	0.465	1.592	-8.403 to 11.586	0.751	-47.402	-84.814 to -9.990	0.014
Anthracyclines	-0.563	-12.490 to 11.365	0.925	0.264	-1.676 to 2.203	0.787	2.700	-7.873 to 13.273	0.612	12.411	-28.476 to 53.299	0.547
Doxorubicin	4.164	-6.549 to 14.878	0.441	0.388	-1.334 to 2.111	0.654	4.825	-4.761 to 14.412	0.319	-24.644	-61.004 to 11.717	0.181
Epirubicin	-4.150	-14.782 to 6.482	0.439	-0.069	-1.781 to 1.643	0.936	-2.518	-12.082 to 7.046	0.601	32.670	-3.037 to 68.377	0.072
Cumulative dose	-0.026	-0.092 to 0.041	0.441	0.007	-0.003 to 0.017	0.187	-0.032	-0.093 to 0.029	0.303	-0.100	-0.356 to 0.155	0.434
Anti-HER												
Trastuzumab	-3.776	-21.187 to 13.636	0.667	-0.687	-3.519 to 2.145	0.630	-3.709	-19.166 to 11.747	0.634	7.749	-52.140 to 67.637	0.797
Pertuzumab	-2.256	-12.996 to 8.484	0.676	-0.125	-1.874 to 1.624	0.887	-1.498	-11.040 to 8.044	0.755	10.969	-25.895 to 47.833	0.555
TDM-1	-1.775	-16.125 to 12.576	0.806	1.328	-0.986 to 3.642	0.256	-5.944	-18.614 to 6.725	0.353	-32.065	-80.809 to 16.679	0.194
Cyclophosphamide	-1.170	-13.314 to 10.974	0.848	0.463	-1.481 to 2.408	0.636	2.644	-8.239 to 13.527	0.629	7.475	-34.076 to 49.025	0.721
Taxane	-14.174	-36.468 to 8.121	0.209	0.789	-2.875 to 4.453	0.669	-13.524	-33.286 to 6.239	0.177	-1.437	-78.931 to 76.056	0.971

Radiation therapy	-6.242	-18.077 to 5.994	0.296	-1.520	-3.427 to 0.386	0.116	-2.560	-13.134 to 8.015	0.631	20.106	-20.607 to 60.819	0.328
Chemotherapy time (months)	-1.223	-3.350 to 0.904	0.255	0.047	-0.310 to 0.404	0.794	-1.143	-3.053 to 0.768	0.237	-2.930	-10.427to4.568	0.438
Anti-HER time (months)	-0.029	-0.262 to 0.203	0.801	0.010	-0.029 to 0.048	0.623	-0.066	-0.272 to 0.140	0.522	-0.334	-1.140 to 0.471	0.410
2D parameters												
LV end-diastolic volume (ml)	-0.220	-0.483 to 0.043	0.099	-0.034	-0.077 to 0.009	0.118	-0.171	-0.406 to 0.064	0.150	0.626	-0.287 to 1.539	0.176
LV end-systolic volume (ml)	-0.203	-0.723 to 0.316	0.438	-0.062	-0.146 to 0.022	0.144	-0.234	-0.694 to 0.225	0.313	0.906	-0.873 to 2.685	0.313
LVEF (%)	-0.338	-1.031 to 0.355	0.334	0.022	-0.092 to 0.135	0.703	-0.032	-0.652 to 0.588	0.918	-0.127	-2.528 to 2.274	0.916
GLS (%)	2.054	0.155 to 3.952	0.034	-0.006	-0.329 to 0.317	0.969	1.031	-0.696 to 2.759	0.237	-5.588	-11.494 to 0.319	0.063
LV stroke volume (ml)	-0.384	-0.847 to 0.079	0.101	-0.011	-0.088 to 0.066	0.769	-0.378	-0.771 to 0.015	0.059	0.283	-1.012 to 1.578	0.660
LV cardiac output (l/min)	-1.348	-5.730 to 3.035	0.537	-0.363	-1.056 to 0.330	0.295	-0.883	-4.652 to 2.885	0.637	-0.378	-0.771 to 0.015	0.059
LA diameter (mm)	-0.360	-1.525 to 0.804	0.539	-0.145	-0.332 to 0.041	0.125	-0.114	-1.1.51 to 0.924	0.828	2.360	-1.604 to 6.324	0.239
LA volume (ml)	**-0.380**	-0.726 to -0.034	0.032	-0.039	-0.099 to 0.020	0.189	-0.356	-0.676 to 0.037	0.029	0.277	-1.070 to 1.624	0.682
Transmitral E/A ratio	-10.174	-21.737 to 1.389	0.084	-0.458	-2.384 to 1.468	0.637	-12.015	-22.0 to 2.029	0.019	0.317	-40.240 to 40.873	0.988
Mitral E/e' ratio	1.799	-0.448 to 4.047	0.114	0.069	-0.301 to 0.439	0.711	1.637	-0.287 to 3.561	0.094	-2.257	-9.161 to 4.647	0.516
TAPSE (mm)	-1.761	-3.221 to -0.301	0.119	-0.056	-0.300 to 0.189	0.651	-1.052	-2.388 to 0.285	0.121	3.509	-1.617 to 8.636	0.176
Tricuspid S' (cm/s)	-0.892	-3.219 to 1.435	0.446	0.221	-0.151 to 0.594	0.239	-0.794	-2.814 to 1.227	0.435	2.122	-5.853 to 10.097	0.596
3D parameters												
LV end-diastolic volume (ml)	-0.327	-0.802 to 0.147	0.168	-0.025	-0.087 to 0.038	0.430	-0.243	-0.651 to 0.165	0.232	0.406	-1.156 to 1.967	0.598
LV end-systolic volume (ml)	-0.397	-1.339 to 0.546	0.395	-0.018	-0.141 to 0.106	0.770	-0.334	-1.136 to 0.468	0.400	-0.378	-3.420 to 2.664	0.801
LVEF (%)	-0.537	-2.059 to 0.986	0.475	-0.048	-0.246 to 0.150	0.623	-0.256	-1.56 to 1.048	0.690	2.894	-1.863 to 7.652	0.222

GWI: global work index, GWE: global work efficiency, GCW: global constructive work, GWW: global wasted work, CI: confidence interval, ACE: Angiotensin-converting enzyme, ARB: Angiotensin II receptor blocker, HER2: human epidermal growth factor receptor, TDM-1: Trastuzumab emtansine, 2D: two-dimensional, LV: left ventricle, LVEF: left ventricle ejection fraction, GLS: global longitudinal strain, LA: left atrium, 3D: three-dimensional.
